# Advances in the Molecular Mechanisms of Pulmonary Fibrosis in Systemic Sclerosis: A Comprehensive Review

**DOI:** 10.3390/ijms262010103

**Published:** 2025-10-17

**Authors:** María Pilar Iranzo Alcolea, Grisell Starita Fajardo, Mercedes Peña Rodríguez, David Lucena López, Cecilia Suárez Carantoña, María López Paraja, Ana García de Vicente, Adrián Viteri-Noël, Andrés González García

**Affiliations:** 1 Systemic Autoinmmune Diseases Unit, Department of Internal Medicine, Hospital Universitario Ramón y Cajal, IRYCIS, 28034 Madrid, Spain; mariapilar.iranzo@salud.madrid.org (M.P.I.A.); grisell.starita@salud.madrid.org (G.S.F.); mpenar@salud.madrid.org (M.P.R.); david.lucena@salud.madrid.org (D.L.L.); mlparaja@salud.madrid.org (M.L.P.);; 2 Department of Radiology, Hospital Universitario Ramón y Cajal, IRYCIS, 28034 Madrid, Spain; ana.garciav@salud.madrid.org

**Keywords:** systemic sclerosis, interstitial lung disease, fibrosis

## Abstract

This document provides an updated overview of the molecular mechanisms underlying pulmonary fibrosis associated with Systemic Sclerosis (SSc). It summarizes current knowledge on how immune activation, vascular injury, and impaired tissue repair contribute to interstitial lung disease (ILD), which is the most serious and life-threatening complication of SSc. SSc is a rare autoimmune disorder involving vascular dysfunction and progressive fibrosis of the skin and internal organs. In the lungs, the interaction between immune and vascular abnormalities and excessive extracellular matrix deposition leads to irreversible structural damage. These processes occur through complex, multifactorial mechanisms that are only partially understood. The review examines recent evidence on the cellular mediators, signaling pathways, and epigenetic alterations involved in ILD-SSc pathogenesis. It also discusses the potential roles of genetic predisposition, environmental factors, and autoantibody profiles in disease heterogeneity. Finally, it highlights emerging therapeutic strategies that target these molecular mechanisms. This work aims to integrate these advances to provide a clearer understanding of the biological basis of SSc-associated pulmonary fibrosis and support the development of novel diagnostic and therapeutic approaches that may improve patient outcomes.

## 1. Introduction

Systemic Sclerosis, also known as scleroderma (SSc), is a rare multisystem connective tissue disease characterized by vascular dysfunction, skin and organ fibrosis, and autoantibodies against various cellular antigens. First described by Carlo Curzio in the mid-18th century, it was initially recognized as a condition that caused the skin to become rigid and resulted in the rapid death of those affected [1]. It is a complex disease with the highest morbidity and mortality rates among autoimmune diseases [2].

The incidence and prevalence of the disease vary greatly depending on the study. Incidence is estimated to range from eight to 56 new cases per million people per year, while prevalence ranges from 28 to 341 per million people. Women are affected three times more often than men and black patients tend to experience an earlier onset with more severe clinical manifestations, as well as a higher risk of pulmonary involvement and scleroderma renal crises [3].

The pathogenesis of the disease is complex and not fully yet understood. Key factors include immunological activation, vascular damage, and excessive extracellular matrix synthesis with collagen deposition [4]. One of its most serious and frequent complications is interstitial lung disease (ILD), one of the leading causes of morbidity and mortality in these patients. ILD in SSc (ILD-SSc) develops as a result of a complex interaction between tissue damage, persistent immune activation, and the dysregulation of repair mechanisms. Over time, this leads to progressive pulmonary fibrosis. In recent years, research has identified multiple cellular and molecular pathways involved in the pathogenesis, including profibrotic mediators, cytokines, extracellular matrix alterations, and epigenetic mechanisms [5].

The diagnosis and monitoring of SSc is complex. It must integrate symptoms, physical examination, laboratory analysis, and imaging tests. In this context, capillaroscopy has become an essential technique in the diagnosis of SSc. It allows direct, non-invasive observation of the capillaries at the base of the nails. Microvascular alterations characteristics of this disease are evident there. The presence of certain patterns helps to distinguish SSc from other connective tissue disorders and to identify the disease in its early stages [6].

On the other hand, lung ultrasound (LUS) has become a reliable diagnostic tool for early detection of interstitial lung disease [7]. The identification and quantification of ultrasound artifacts such as B-lines or pleural thickening/irregularity allowed for an indirect assessment of the degree of interstitial involvement. Several studies have demonstrated a significant correlation between ultrasound findings and results from high-resolution computed tomography (HRCT) [8]. Further studies are needed, but LUS is positioned as a useful complementary tool for the clinical follow-up and monitoring of lung involvement progression in these patients.

Improving diagnosis and prognosis, and developing more effective targeted therapies, requires an understanding of these processes at the molecular level. This review highlights the main molecular mechanisms underlying in ILD-SSc development and progression, emphasizing signaling pathways, effector cells, and mediators that could be emerging therapeutic targets.

## 2. General Pathophysiology of ILD in SSc

ILD is currently the leading cause of morbidity and mortality in patients with SSc. It accounts for 35% of disease-related and 20% of overall mortality in these patients [1]. Up to 80% of patients with SSc have imaging results consistent with ILD on HRCT. However, only 30–40% of these patients will develop clinically significant disease [5].

Firstly, it must be emphasized that the pathogenesis of ILD-SS is not fully understood [9]. It is believed to result from a complex network of interactions involving genetic predisposition, immune dysregulation, and as yet undetermined environmental factors. Genetic factors, such as Human Leukocyte Antigen (HLA) haplotypes and polymorphisms in immune system regulatory genes, may predispose individuals to abnormal immune responses. Following exposure to environmental triggers, which may include viral infections, inorganic dusts, or organic solvents, abnormal and persistent immune activation may be experienced by these genetically susceptible individuals, leading to excessive autoantibody production and chronic inflammation [10]. These factors ultimately drive aberrant fibroblast activation, leading to excessive extracellular matrix deposition and progressive fibrosis. At the same time, vascular damage and endothelial dysfunction would worsen tissue hypoxia, further increasing fibrotic remodeling with the activation of various molecular pathways that will be discussed throughout this article.

The risk of developing ILD increases over the course of the disease. Nevertheless, this complication typically manifests during the first five years after the onset of the first symptoms.

### 2.1. Relationship Between Genetics and Fibrosis

The pathophysiology of ILD-SSc is still unknown, but it is hypothesized that there may be an interrelationship between autoimmunity, fibrosis, inflammation, and vascular damage. The molecular mechanisms involved in the clinical and pathologic manifestations of the disease are highly complex, and although numerous studies have provided substantial information about its intricate picture and clarified some of its early events, the precise altered regulatory pathways involved have not been completely elucidated [9].

All these processes are likely guided by a genetic substrate. Some type of damage to the alveolar epithelium or the surrounding vasculature has been proposed as a potential triggering event. Some hypotheses suggest the possible involvement of a viral trigger. This has become more relevant following the detection of early type I interferon axis activation, as well as Toll-like receptor 8 (TLR8) activation by Epstein–Barr Virus (EBV) genes in monocytes from these patients [10]. In this context monocytes and T lymphocytes have been proposed as the main activators of the cascade of initial damage and subsequent fibrosis, involving multiple molecular alterations that could occur simultaneously or sequentially [9]. These interconnected mechanisms ultimately manifest in a wide range of pathological features, including:

- Fibroproliferative lesions of small arteries and arterioles are accompanied by severe structural and functional alterations in endothelial cells.
- Severe oxidative stress and elevated levels of reactive oxygen species (ROS).
- Excessive and often progressive deposition of collagen and other extracellular matrix (ECM) macromolecules.
- Alterations in cellular and humoral immunity with the production of numerous autoantibodies.
- Cell transdifferentiation occurs with the phenotypic conversion of various cell types, such as resting fibroblasts, endothelial cells, epithelial cells, and adipocytes, into activated myofibroblasts.
- Excessive production and release of various cytokines and growth factors with profibrotic and inflammatory effects.
- Epigenetic alterations, including numerous changes mediated by non-coding RNA.

#### 2.1.1. Genetic Factors

Family clustering, a high prevalence of various autoimmune diseases among relatives, and the phenotypic differences observed among ethnic groups suggest the involvement of genetic factors [11]. While SSc is not considered a genetic or hereditary disease, numerous genetic variants, both within and outside the HLA complex, have been associated with an increased risk of developing SSc [12].

##### HLA Genes

The HLA-coding genes are located on chromosome 6 and are highly polymorphic. HLA-II genes have been more frequently associated with SSc than HLA-I genes. The most frequently found alleles are HLA-DRB1, HLA-DQB1, HLA-DPB1, and HLA-DOA1. Note that these HLA associations may be common in different ethnic groups while others are specific to certain populations [13,14]. The HLA-DRB1*11 haplotype, specifically HLA-DRB1*1104, has been shown to increase susceptibility to SSc. This correlation has also been observed between the HLA-DQB1*0501 haplotype and anti-topoisomerase antibodies (Anti-Scl-70). Conversely, several alleles (HLA-DRB1*1501, DRB1*0701, DQA1*0102, and DQB1*0602) have been identified that may protect against SSc; these alleles are less common in patients with the disease.

##### Non-HLA Genes

In addition to HLA genes, multiple non-HLA genetic variants associated with SSc susceptibility have been identified. These variants are located on different chromosomes and are involved in various biological processes, such as innate and adaptive immunity, cell signaling, cytokine production, extracellular matrix formation and deposition, apoptosis, and autophagy.

Within the Major Histocompatibility Complex (MHC) region, two relevant non-HLA genes have been identified: PSORS1C1 [15], whose specific function is still unknown, but which has been linked to other autoimmune diseases, including Systemic Lupus Erythematosus (SLE) [16], rheumatoid arthritis, and Behçet’s disease [17]; and NOTCH4 [18,19], which encodes a transmembrane receptor involved in the regulation of fibrosis and vascular function. The role of Notch signaling in the regulation of EMT is suggested by indirect and direct studies. Notch signaling molecules are reported to activate directly transforming growth factor beta (TGFβ) in rat mesangial cells under hyperglycemic conditions [20]. In addition to these direct effects mediated by its intracellular domain, Notch can indirectly regulate EMT through other signaling pathways, including TGFβ [21], Nuclear Factor kappa-light-chain-enhancer of activated B cells NF-κB [22] and β-catenin [23], and through the action of various regulatory miRNAs [24,25,26,27,28].

Numerous variants have additionally been identified in key genes located outside the HLA region, including IRF5, STAT4, CD247, BANK1, and BLK [13,29,30,31]. These genes are functionally relevant due to their involvement in the immune response. IRF (Inteferon regulatory factor) genes activate type I interferon [32], which is significantly increased in patients with SSc; STAT4 and CD247 regulate key T lymphocyte functions, while BANK1 and BLK are related to B lymphocyte activation and signaling. In animal models, STAT4 deficiency has been shown to reduce cytokine production and protect against fibrosis [33].

Similarly, genes related to the tumor necrosis factor alpha pathway (TNFAIP3, TNIP1, and TNFSF4/OX40L) [34,35,36] and interleukin 12 signaling (IL12A, IL12RB1, IL12RB2, TYK2, and STAT4) have been identified [30,37,38,39,40]. STAT4 polymorphisms can alter IL-12 signaling and influence fibrosis risk by affecting the activation and function of STAT4. Such polymorphisms may affect phosphorylation, nuclear translocation, and transcriptional activation of target genes. In consequence STAT4 function can shift the balance away from protective Th1 immune responses towards other immune phenotypes (e.g., Th2 or Th17), which are linked to increased fibrosis risk [41,42]. Recent studies have also begun exploring the interaction between genetic and environmental factors. One notable example is the interaction between the rs58905141 polymorphism in TNFAIP3 and exposure to silica particles [43], which induces a fibroinflammatory response in cellular models. Taken together, these findings suggest that the pathogenesis of SSc results from a complex interaction between genetic susceptibility and environmental factors.

#### 2.1.2. Fibrosis

During the fibrotic process, connective tissue is deposited in the extracellular matrix, which progressively replaces normal tissue architecture. This process is usually accompanied by vascular remodeling of small arteries and arterioles [44]. The initial stimulus that triggers this process remains unknown. However, numerous cytokines and growth factors, such as interleukin 1 (IL-1), IL-4, IL-6, IL-8, IL-13, IL-17, TGF-β, interferon gamma, platelet-derived growth factor (PDGF), and tumor necrosis factor (TNF) [45], are involved.

Fibroblasts in patients with SSc have an inherent microfibril defect containing fibrillin-1 and continuously overexpress the type I collagen gene autonomously [46]. Isoform 1 plays an important role in fibrosis because it is an indirect mitogen of fibroblasts; meanwhile, isoforms 2 and 3 appear to exert a regulatory role in TGF-β activation, particularly isoform 2 as isoform 3 is almost negligible outside the embryonic period. They do not directly promote the transition towards activated fibroblasts, but their involvement suggests that they have a modulatory function in this process [47,48]. While the pathogenesis of fibrosis remains poorly understood, it is clear that the multifunctional cytokine TGF-β is essential to the process [49], promoting the activation of fibroblasts and their transformation into myofibroblasts. This increases the synthesis of the extracellular matrix, including type I and III collagen, fibronectin, and tenascin-C, while inhibiting its degradation.

It is important to note that the pathogenic fibrotic process in ILD-SSc shares mechanical parallels with idiopathic pulmonary fibrosis (IPF) in terms of epithelial injury and fibroblast activation via epithelial–mesenchymal transition (EMT) and endothelial-mesenchymal transition (EndoMT) [50]. Studies show that EMT is regulated by pathways such as TGF-β1/Smad, and modulating this pathway can affect the progression of fibrosis in SSc-ILD. Also, aberrant basaloid cells expressing epithelial–mesenchymal markers have been identified in SSc-ILD lungs, indicating EMT occurrence [51]. In patient lung tissue, EndoMT contributes to vascular remodeling and fibrosis as endothelial cells acquire mesenchymal features, while EMT drives alveolar epithelial cells to become myofibroblasts that produce excessive ECM [52].

### 2.2. Differences from Other ILD

Initially, ILD associated with SSc was considered indistinguishable from Idiopathic Pulmonary Fibrosis (IPF). Before the Katzenstein and Myers classification was published in 2001 [53], it was thought that the two conditions had identical histologies, though ILD associated with SSc tended to have a better prognosis than IPF.

Currently, Interstitial Lung Diseases (ILD) are classified according to etiology and histological pattern. ILD-SSc associated pneumopathy usually affects women between the ages of 30 and 60, whereas IPF is more prevalent in men over 60 years old [54].

**Figure 1:**
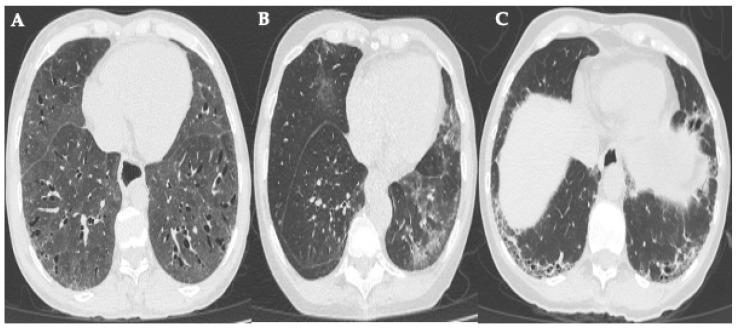
High-resolution axial computed tomography images with different patterns of ILD (Source: Radiology department of Ramon y Cajal’s Hospital). The images show patterns of non-specific fibrotic interstitial pneumonia (**A**,**B**), highlighting the presence of ground-glass opacities and traction bronchiectasis. Image (**C**) shows usual interstitial pneumonia, where the previous findings and a pattern of honeycombing can be seen. The dilated esophagus suggests ILD related to connective tissue disease rather than idiopathic pulmonary fibrosis.

From a radiological point of view, HRCT has revolutionized the diagnosis and follow-up of these patients by improving the accuracy with which the different patterns can be identified and described. Non-specific interstitial pneumonia (NSIP) associated with SSc is characterized by more homogeneous involvement of the pulmonary interstitium. In contrast, IPF typically involves subpleural fibrosis, reticularity, and honeycombing [54]. Figure 1 shows CT images with different patterns of interstitial lung involvement.

At the histological level, IPF is associated with the pattern of Usual Interstitial Pneumonia (UIP), while ILD-SSc typically presents a pattern of Non-Specific Interstitial Pneumonia (NSIP). UPI is characterized by a heterogeneous damage distribution, with alternating areas of normal lung parenchyma, dense fibrosis, honeycombing, and active fibroblastic foci, reflecting an aberrant and progressive repair process. In contrast, NSIP shows a more homogeneous interstitial involvement, with uniform fibrosis of mild to moderate intensity and a diffuse interstitial inflammation, predominantly lymphoplasmacytic. In NSIP, the alveolar architecture is usually relatively preserved, and honeycombing is rare, contributing to a better prognosis and greater response to immunosuppressive treatment compared to UIP [55]. Histological diagnosis is key, given that the distinction between the two patterns has prognostic and therapeutic implications. However, this distinction can be challenging, and both patterns may be present in biopsies from the same patient [56].

### 2.3. Role of Autoantibodies

The formation of autoantibodies plays a key role in ILD-SSc. The initial stimulus for their production is unknown. Between 75% and 90% of patients test positive for antinuclear antibodies (ANAs), and some studies report figures above 90% [57].

Each patient with SSc normally produces a single type of autoantibody that serves as a biomarker for different patterns of skin and visceral involvement and as a prognostic indicator. For example, anticentromere antibodies (ACA) and antitopoisomerase antibodies (Anti-Scl-70) are generally mutually exclusive [58,59], and they are identified simultaneously in only 0.5% of patients.

Anti-Scl-70 antibodies are most strongly associated with the development and severity of ILD-SSc. However, the search for new biomarkers is ongoing. Approximately 10% of patients with “seronegative” SSc have been found to have specific antibodies, including anti-elF2B, anti-RuvBL1/2 complex, anti-RNP U11/U12, anti-RNPC3 and anti-BICD2. These antibodies are associated with serious organ complications [4,59,60,61,62,63,64,65].

In recent years, however, new evidence has emerged regarding antibodies such as anti-Ro52. Anti-Ro52 has been linked to progressive lung involvement regardless of Anti-Scl-70 positivity and is being proposed as a new biomarker of disease aggressiveness. Another antibody, anti-citrullinated peptide (anti-CCP) [66], has been linked to a higher incidence and severity of ILD-SSc, especially with the histological pattern of NIU and lower diffusion capacity (DLCO) values. However, its predictive value in this regard is not well established.

Finally, some very rare autoantibodies have been identified in small subgroups of patients. These include those directed against eukaryotic translation initiation factor 2B (eIF2B) and telomere-associated proteins, such as TERF1 [67]. Their presence may correlate with more severe forms of lung involvement and/or a clinically more rapid and aggressive course. Table 1 summarizes the autoantibodies, their frequencies, and their most frequently associated clinical manifestations.

## 3. Key Cells Involved

ILD progression does not depend exclusively on initial inflammation or adaptive autoimmunity. It also depends on sustained dysregulation of structural lung parenchyma cells and the innate immune system. This results in the persistent activation of signaling pathways that induce tissue remodeling and aberrant ECM deposition. The cells most involved in this process are listed below.

### 3.1. Alveolar and Interstitial Macrophages

Macrophages are versatile cells that demonstrate great plasticity depending on their tissue microenvironment. Traditionally, macrophage activation has been classified into two categories: the proinflammatory M1 pathway and the profibrotic M2 pathway. Though limited, this classification remains useful for understanding their role in fibrosis.

In ILD-SSc, macrophages exhibit characteristics of both M1 and M2, with a predominance of M2 polarization. This polarization is associated with increased production of profibrotic mediators and poor inflammation resolution [68]. Additionally, macrophages in ILD-SSc exhibit impaired efferocytosis (the defective removal of apoptotic cells), particularly when levels of CXCL4 (a platelet-activating chemokine) are elevated. This results in poor clearance of dead cells and perpetuates the inflammatory state [69]. Taking this into account, CXCL4 is emerging as a biomarker for the onset and progression of SSc. On the other hand, increased expression of Fcγ receptors in pulmonary macrophages enhances their phagocytic and inflammatory responses, adding another pathway of tissue damage.

Finally, a significant molecular-level difference should be noted. While the MAP kinase (MAPK) signaling pathway is activated in alveolar macrophages in ILD-SSc, Th17 signaling predominates in IPF [70]. Both mechanisms are still under study.

### 3.2. Type I and II Pneumocytes

Pneumocytes are specialized epithelial cells that line the alveoli of the lung and are essential for gas exchange and alveolar homeostasis [71]. There are two main types:

- **Type I pneumocytes:** Large flat cells cover approximately 95% of the alveolar surface and facilitate gas diffusion. Injury to type I pneumocytes in ILD-SSc disrupts the alveolar-capillary barrier leading to increased permeability, exposure of the underlying basement membrane, and release of damage-associated molecular patterns. This injury initiates local inflammation and promotes the recruitment, differentiation, activation and proliferation of fibroblasts and myofibroblasts, contributing to the fibrotic cascade [72].
- **Type II pneumocytes:** Cuboidal cells responsible for synthesizing and secreting pulmonary surfactant. They serve as progenitor cells that can proliferate and differentiate into type I pneumocytes. Histopathological studies show type II pneumocyte hyperplasia ILD-SSc lung biopsies [71]. In ILS-SSc, repeated injury and chronic inflammation drive type II pneumocytes to undergo aberrant activation and senescence. Senescent type II pneumocytes lose their regenerative capacity and secrete profibrotic mediators, such as TGF-β, which further stimulate fibroblast proliferation and myofibroblast differentiation, perpetuating fibrosis [73]. The loss of normal type II pneumocyte function impairs alveolar regeneration and shifts the microenvironment toward persistent extracellular matrix deposition [74].

### 3.3. Activated Fibroblasts and Myofibroblasts

Fibroblasts are mesenchymal cells found in connective tissue that participate in tissue repair and maintenance of the ECM under normal conditions. In ILD-SSc, these fibroblasts undergo persistent activation through profibrotic cytokines, notably TGF-β, differentiating into myofibroblasts. Myofibroblasts are highly secretory, contractile cells that express markers such as α-SMA (α-smooth muscle actin). This promotes increased deposition of collagen and other ECM proteins, favoring distortion of the normal lung parenchyma architecture [75].

Single-cell transcriptomic studies, which allow researchers to study genes expressed in individual cells, have revealed significant heterogeneity among fibroblast populations in the lungs of patients with ILD-SSc [75]. Not only are myofibroblasts more abundant in these patients, but they also show greater resistance to apoptosis, like macrophages.

### 3.4. T and B Lymphocytes

T and B lymphocytes play a key role in initiating and perpetuating the fibrotic process. TCD4+ lymphocytes infiltrate lung tissue and differentiate into Th2 phenotypes, producing IL-4, IL-13, and IL-17, as well as Th17. These promote the activation of M2 macrophages [76].

B lymphocytes, on the other hand, become abnormally activated. They produce autoantibodies and cytokines, such as IL-6 and TGF-β. These cytokines promote a profibrotic microenvironment and in turn, they induce the activation of T cells and fibroblasts. Additionally, B lymphocytes act as antigen-presenting cells, thereby reinforcing the adaptive immune response. Clinical studies have shown that the use of anti-CD20 therapies (such as rituximab) stabilizes or improves lung function in patients with ILD-SSc, supporting the direct involvement of B lymphocytes in its pathogenesis [77]. Taken together, these immune cells not only participate in early inflammation, but also contribute to the establishment and persistence of pulmonary fibrosis.

## 4. Signaling Pathways and Molecular Mediators

**Figure 2:**
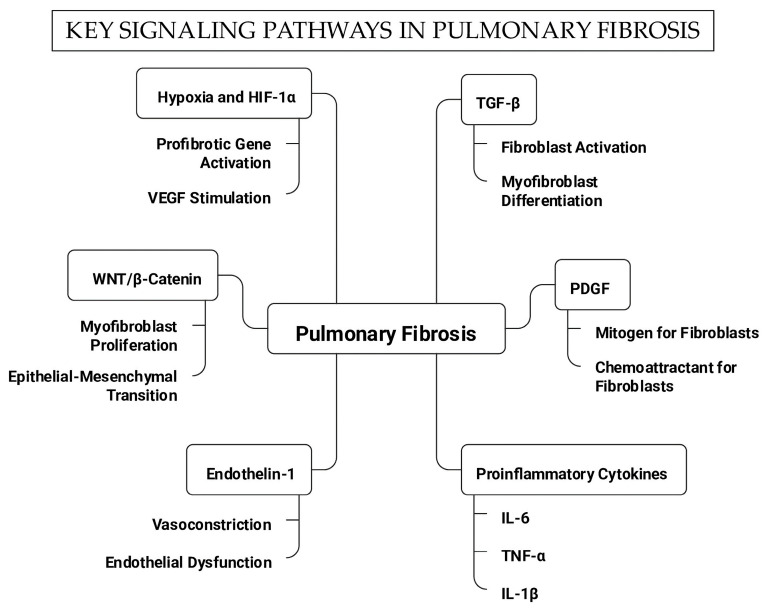
Key mechanisms in ILD-SSc (created by the authors, based on data from references [79,80,81,82,83,84,85,86,87,88,89,90,91,92,93,94,95,96,97,98,99,100,101,102,103,104,105,106,107,108,109,110,111,112]).

The process of pulmonary fibrosis in SSc is not linear. Rather, it involves a dynamic interaction between inflammation, endothelial dysfunction, and profibrotic signaling. Various signaling pathways and molecular mediators have been identified as key players in this phenomenon. Characterizing them has been essential for understanding the disease’s underlying mechanisms and promoting the development of targeted therapies. Some of the most important pathways identified are detailed below. Figure 2 shows a scheme integrating the different key mechanisms.

### 4.1. TGF-β (Transforming Growth Factor Beta)

TGF-β is the most studied and central pathway in fibrosis. It is the primary molecular driver of fibroblast and myofibroblast activation and differentiation in ILD-SSc [78].

The three TGF-β isoforms (TGF-β1, TGF-β2, and TGF-β3) are key regulators of cell differentiation, migration, proliferation, and gene expression. They have been associated with reparative and fibrotic responses. The TGF-β signaling cascade remains active in fibrotic tissues regardless of the initial injury’s etiology [78].

Multiple mediators, such as angiotensin II and norepinephrine, stimulate TGF-β transcription [79]. Additionally, oxidative stress, signaling through Toll-like receptors (TLRs), and proinflammatory cytokines, such as IL-1β, TNF-α, and IL-6, have been shown to induce TGF-β isoforms transcription in fibrotic tissues.

The fibrogenic actions of TGF-β are primarily attributed to its essential role in converting fibroblasts into myofibroblasts, which is a defining characteristic of tissue fibrosis. Compared to myofibroblasts located in normal tissues, these activated myofibroblasts in fibrotic lesions show increased expression of structural collagens capable of synthesizing a wide variety of extracellular matrix proteins, proinflammatory cytokines, and growth factors [80].

The effects of TGF-β on fibroblast proliferation are inconsistent, with both pro- and anti-proliferative actions having been described. These discrepancies may highlight the context-dependent nature of TGF-β, as well as the functional and phenotypic heterogeneity of fibroblast populations, depending on additional mediators, cell differentiation status, and extracellular matrix environment [81].

Conversely, TGF-β promotes matrix preservation by suppressing protease activity through the induction of inhibitors, such as plasminogen activator inhibitor-1 (PAI-1) and tissue metalloprotease inhibitors (TIMPs) [82].

It should be noted that some of the profibrotic effects of TGF-β may not result from the growth factor’s direct actions, but rather from its ability to induce the secretion of other fibrogenic factors. For example, TGF-β-induced IL-11 expression has been identified as a critical fibrogenic signal that acts through activation of the Erk pathway [83]. Other research has suggested that CCN2, a matrix protein also known as connective tissue growth factor (CTGF), is a key TGF-β-induced signal responsible for myofibroblast conversion and fibrogenic signaling. This is based on experiments requiring the co-administration of CCN2 and TGF-β in subcutaneous tissue to achieve sustained fibrosis [84], although this possible signaling pathway remains controversial [85].

The repair response to many types of injuries relies on the activation of the TGF-β cascade, but this must be tightly regulated. Therefore, chronic fibrotic conditions may result from a lack of endogenous negative regulation. Signals that restrict TGF-β signaling can act at various levels. Notable among these are the inhibitory Smads (I-Smads, or Smad6 and Smad7), which form a TGF-β-induced subfamily that antagonizes TGF-β signaling. Furthermore, several other signals can indirectly suppress TGF-β signaling by interfering with its synthesis or modulating cascades that interact with the TGF-β/R-Smad axis [78].

### 4.2. PDGF (Platelet-Derived Growth Factor)

Platelet-derived growth factor (PDGF) is a profibrotic mediator produced by numerous lung cells, including activated alveolar macrophages, endothelial cells, and fibroblasts. PDGF acts primarily through its receptors, PDGFR-α and PDGFR-β, which are expressed on mesenchymal cells.

In ILD-SSc, both PDGF ligands and receptors are overexpressed in lung tissue and elevated levels of PDGF have been detected in the bronchoalveolar lavage fluid of affected patients, which correlates with increased proliferation and activity of fibroblasts and myofibroblasts [86,87,88]. PDGF acts as a potent mitogen and chemoattractant for fibroblasts, promoting their proliferation, migration, survival, and differentiation into myofibroblasts.

There is a synergistic interaction between PDGF and TGF-β signaling. TGF-β increases PDGFR-α expression in fibroblasts from patients with ES, increasing their sensitivity to PDGF and amplifying the fibrogenic response. This positive feedback loop sustains persistent fibroblast activation and myofibroblast survival [89]. Currently, inhibiting the PDGF/PDGFR axis is an active area of research for developing antifibrotic therapies for ILD-SSc [90].

### 4.3. Proinflammatory Cytokines

Proinflammatory cytokines, such as IL-6, TNF-α and IL-1β amplify the immune response after an initial attack and prepare tissue for the transition to fibrosis.

- **IL-6:** IL-6 is one of the most important proinflammatory cytokines in the early stages of fibrosis [91]. Three important mechanisms have been described thus far:
  - **Activation of fibroblasts via STAT3:** IL-6 promotes the activation of fibroblasts and their differentiation into myofibroblasts, mainly through the Janus Kinase JAK2/STAT3 signaling pathway. This pathway increases the production of type I collagen and other extracellular matrix components [92]. Inhibiting IL-6 or the STAT3 pathway reduces fibroblast proliferation, differentiation into myofibroblasts, and extracellular matrix production. This suggests a direct role in perpetuating fibrosis [93].
  - **Th17 lymphocyte differentiation:** Together with TGF-β, IL-6 promotes the differentiation of CD4+ T cells into the Th17 subtype, which produces IL-17, a cytokine with potent proinflammatory effects [93,94].
  - **B cell activation:** IL-6 stimulates the maturation and activation of B cells, promoting the production of autoantibodies [95].

TGF-β promotes as well as prolongs STAT-3 activation induced by IL-6 and IL-21 through inhibition of SOCS3 expression. The inhibition of SOCS3 by TGF-β releases the negative regulation of STAT3 activation by SOCS3 and result in enhanced STAT3 activation and Th17 cell differentiation. This cross-talk between TGF-β and the downstream signaling molecules of IL-6 and IL-21 plays a crucial role for Th17 cell development and could be at least one of the mechanisms by which TGF-β affects Th17 cell differentiation [96].

- **TNF-α (Tumor Necrosis Factor alpha):** Serum levels of TNF-α are elevated in patients with SSc, particularly those with pulmonary fibrosis. Thise elevated TNF-α levels had been correlated with decreased lung capacity and the presence of fibrosis. In 1997 Hasegawa, M et al. demonstrated that serum TNF-α levels were elevated in patients with SSc and correlated with the presence of pulmonary fibrosis and with the decreased vital capacity of the patients [91]. This has also been demonstrated in animal models, with motheaten mutant mice exhibiting rapid progression of pulmonary fibrosis due to elevated levels of TNF-α in both serum and lungs [97]. Additionally, TNF-α induces IL-6 and CCL2 secretion by systemic sclerosis fibroblasts, thereby enhancing their activation and differentiation into myofibroblasts perpetuating inflammation and fibrosis [98]. In summary, TNF-α, IL-17, IL-6 and TGF-β form an interactive cytokine network which amplifies inflammation and regulates fibroblast activation and fibrogenesis. Within this network, TNF-α modulates IL-17 production and signaling, thereby potentiating the intensity of the inflammatory response and the dynamics of tissue remodeling together with IL-6 and TGF-β. The final outcome depends on the cellular context and the balance between the signaling pathways involved [99].
- **IL-1β:** Promotes the expression of metalloproteinases (MMPs), which remodel the extracellular matrix. It also promotes the production of IL-6, IL-17, and TNF-α. However, it has been shown to have direct antifibrotic effects on fibroblasts, highlighting the complexity of its role in the pathogenesis of ILD-SSc [100,101].
- **IL-13:** Although it is more associated with the Th2 axis, IL-13 directly stimulates collagen production by fibroblasts. Additionally, it plays a significant role in promoting the polarization of macrophages to the M2 phenotype [102].
- **IL-17:** IL-17, especially IL-17A, plays a direct proinflammatory and pro-fibrotic role in the pathogenesis of ILD-SSc. It is primarily produced by Th17 cells, which are increased in circulation in SSc and correlate with disease activity and extent of fibrosis [103]. It also promotes the activation and proliferation of lung fibroblasts, leading to increased collagen and extracellular matrix production, stimulates other pro-fibrotic cytokines and mediators, such as TGF-β and connective tissue growth factor (CTGF) ending in further accelerating fibroblast activation and matrix deposition [104,105].

### 4.4. Endothelin-1 (ET-1)

ET-1 is a vasoactive peptide that has potent profibrotic and vasoconstrictive effects. It is primarily produced by endothelial cells and fibroblasts. ET-1 acts through ETA and ETB receptors and has been associated with the vasculopathy and fibrosis that are characteristic of SSc. ET-1 is overexpressed in lung tissue and the bloodstream [96]. Furthermore, ET-1 has been proposed to contribute not only to fibrosis but also to endothelial dysfunction and pulmonary hypertension.

In conclusion, ET-1 integrates and amplifies the signals of multiple profibrotic cytokines, playing a decisive role in the fibrotic process [106].

### 4.5. WNT/β-Catenin

The Wnt/β-catenin signaling pathway is aberrantly activated in ILD-SSc. This activation is essential for TGF-β/Smad2/3-mediated myofibroblast proliferation and the epithelial–mesenchymal transition. Additionally, it modulates the expression of proinflammatory and profibrotic mediators, such as IL-1β and IL-6, in alveolar epithelial cells [94]. The Wnt/β-catenin pathway is also associated with increased fibroblast migration and proliferation. However, it does not directly induce classic profibrotic markers unless it acts in synergy with TGF-β. Interactions with other relevant pathways, such as PDGF, ET-1, TNF-α, and IL-17, have been demonstrated; these pathways all converge on fibroblast activation and differentiation into myofibroblasts [107,108].

### 4.6. Hypoxia and HIF-1α (Hypoxia-Inducible Factor-1α)

Hypoxia and hypoxia-inducible factor 1 alpha (HIF-1α) play a central role in the pathogenesis of pulmonary fibrosis.

HIF-1α is a transcription factor that regulates the cellular response to hypoxia or high levels of oxidative stress [109]. Under normal oxygen conditions, HIF-1α is rapidly degraded; however, under hypoxic conditions, it accumulates in the cell nucleus and activates profibrotic genes, such as COL1A1, fibronectin, and α-SMA. Additionally, HIF-1α stimulates the production and deposition of the extracellular matrix in a synergistic manner with TGF-β [110].

Lastly, HIF-1α stimulates VEGF (vascular endothelial growth factor), consequently contributing to aberrant angiogenesis and dysfunctional vascularization [111]. The following table (Table 2) summarizes all the mechanisms previously explained.

**Table 2:** Summary of the molecular pathways involved in the pulmonary fibrosis (created by the authors, based on data from references [78,79,80,81,82,83,84,85,86,87,88,89,90,91,92,93,94,95,96,97,98,99,100,101,102,103,104,105,106,107,108,109,110,111]).

Pathway	Key Mediators	Key Effects in Pulmonary Fibrosis	References
TGF-β	IL-1β, TNF-α, and IL-6, Smad2/3, angiotensin II, norepinefrine, ROS	Fibroblast activation and myofibroblast differentiation: increased collagen deposition, protease inhibition. Central role regulating other processes	[78,79,80,81,82,83,84,85]
PDGF	PDGFR-α, PDGFR-β	Fibroblast proliferation and migration synergy with TGF-β	[86,87,88,89,90]
PROINFLAMMATORY CYTOKINES	IL-6, IL-1β TNF-**α** IL-13	STAT3-mediated fibroblast activation, Th17 differentiation, autoantibody production, M2 polarization	[91,92,93,94,95,96,97,98,99,100,101,102,103,104,105]
ENDOTHELIN-1 (ET-1)	ET-1 ETS/ETB receptors	Vasoconstriction and vascular remodeling synergy with TGF-β	[106]
WNT/β—CATENIN	β-catenin Wnt ligands	EMT-fibroblast proliferation; potentiates TGF-B-and. IL-5	[95,107,108]
HIPOXIA	HIF-1α	Activates profibrotic genes, such as COL1A1, fibronectin, and α-SMA; activates VEGF	[109,110,111]

Abbreviations: TGF-β (Transforming growth factor beta); TNF-α (Tumor Necrosis Factor alpha); ROS (reactive oxygen species; PDGF (Platelet-derived growth factor); HIF-1α (Hypoxia-inducible factor-1α).

## 5. Epigenetics

Epigenetics is the study of heritable, stable, and reversible modifications to gene expression and function that do not alter the DNA sequence. ILD-SSc does not depend exclusively on genetic or environmental factors, but also on these epigenetic mechanisms, which are becoming increasingly important [112].

These mechanisms include DNA methylation, histone modifications, and non-coding RNAs. They all actively contribute to the regulation of key profibrotic pathways and mediators, such as TGF-β, endothelin-1, Wnt/β-catenin, HIF-1α, IL-6, IL-1β, IL-17, TNF-α, and PDGF.

- **Aberrant DNA methylation:** Global hypomethylation of TCD4+ cells has been observed due to decreased DNA methyltransferase-1 (DNMT1) activity, which implies constant proinflammatory cell activation. At the other extreme, hypermethylation of the FOXP3 promoter has been observed [113]. FOXP3 is essential for the functioning and regulation of regulatory T cells (Tregs) and typically controls the expression of anti-inflammatory genes, such as IL-10 and TGF-β, while inhibiting the expression of proinflammatory genes in effector T cells. Due to this hypermethylation, there is an imbalance between Th17 and Treg cells, which allows for increased production of IL-17. Consequently, there is increased production of IL-6, TNF-α, and TGF-β by fibroblasts and myofibroblasts in the lungs. Taking all this into account, FOXP3 has become a diagnostic marker and a potential therapeutic target for restoring immune balance [114].
- **Histone modifications:** These modifications further modulate chromatin accessibility, thereby influencing the transcription of genes involved in fibroblast activation, extracellular matrix production, profibrotic growth factors, and cytokine response [115,116]. Hyperacetylation of histone H4 and global hypomethylation in B cells from patients with SSc have been associated with cutaneous sclerosis and increased disease activity [117]. These histone modifications promote accessibility to the promotes of multiple key pathways. For example, they potentiate the TGF-β/Smad pathway and its interrelation with the Wnt/β-catenin pathway. They also facilitate the transcription of ET-1 and PDGF and consequently promote the production of proinflammatory cytokines, including IL-6, IL-1β, and TNF-α. Furthermore, they have the capacity to maintain continuous fibroblast activation through the HIF-1α pathway, allowing chromatin to be reconfigured toward hypoxic adaptation genes [109]. Notably, proinflammatory cytokines can trigger epigenetic changes and persistently activate the phenotypic transdifferentiation of pulmonary fibroblasts [118].
- **Non-coding RNAs (ncRNAs):** There has been a growing interest in studying ncRNAs, including microRNAs (miRNAs) and long non-coding RNAs (lncRNAs), in recent years due to their important role in controlling various biological processes.
  - **MicroRNAs (miRNAs):** are small RNA molecules consisting of 22 nucleotides that regulate gene expression post-transcriptionally. They regulate key pathways responsible for fibroblast activation, extracellular matrix remodeling, and TGF-β signaling [119]. In lung fibroblasts, TGF-β alters the miRNA repertoire, driving the transition from fibroblast to myofibroblast [117]. TGF-β-induced miRNAs, such as miR-21-3p and miR-455-3p, then feed back into the TGF-β and Wnt pathways, promoting excessive ECM production. Conversely, the loss of antifibrotic miRNAs prevents proper inhibition of collagen production [120,121]. In ILD-SSc, miR-320a is decreased in both serum and lung. Its restoration has been shown to reduce collagen by targeting TGFBR2 and IGF1R, thereby integrating post-transcriptional control of the TGF-β/PI3K-AKT pathway in fibroblasts [122]. Additionally, studies on pulmonary fibrosis have demonstrated that miR-335-3p attenuates TGF-β activation by suppressing THBS1, a TGF-β activator. This leads to a reduction in fibrotic markers within the respiratory epithelium [123].
  - **Long non-coding RNAs (lncRNAs):** These molecules consist of more than 200 nucleotides and can regulate gene expression at the transcriptional and post-transcriptional levels [124]. They modulate the response to IL-1β in pulmonary fibroblasts through opposing mechanisms. For example, they regulate the response to IL-1β: IL7AS limits IL-6, while MIR3142HG (the precursor of miR-146a) enhances IL-8/CCL2. Dysfunction of MIR3142HG is associated with an altered inflammatory phenotype, connecting lncRNAs with the NF-κB/IL-1β/IL-6 axes [125].

In summary, ncRNAs act as command centers that integrate proinflammatory and profibrotic pathways. The therapeutic modulation of these ncRNAs with inhibitors and analogs is being considered as a possible therapeutic target to deactivate these profibrotic signaling pathways. These therapeutic strategies are under development.

## 6. New Perspectives and Emerging Therapies for the Treatment of ILD-SSc

Comprehending the pathophysiological mechanisms of ILD-SSc is crucial for clinical practice. Recognizing the interplay between immune activation, vascular injury, and fibrosis not only provides insight into disease progression but also helps design therapeutic strategies. For example, identifying early immune-mediated inflammation supports the rationale for immunomodulatory therapies such as rituximab, whereas persistent fibroblast activation underlines the need for antifibrotic agents like nintedanib or nerandomilast. The integration of these mechanistic insights into clinical practice enables a personalized approach, with treatment selection aligned with disease stage and the predominant pathological pathways. Furthermore, emerging therapies for ILD-SSc are increasingly based on and target the key molecular mechanisms involved in the pathogenesis of pulmonary fibrosis. This provides a solid scientific foundation for developing more precise and effective therapeutic strategies. These advances hold great promise for improving outcomes in patients affected by this condition, which remains associated with a very high morbidity and mortality rate [126]. The following list details some of the treatments currently in use, as well as new emerging therapies.

- **Rituximab (RTX):** RTX is a chimeric monoclonal antibody that targets CD20, a surface antigen expressed on pre-B and mature B lymphocytes. By binding to CD20, rituximab induces B-cell depletion through several mechanisms, including complement-dependent cytotoxicity, antibody-dependent cellular cytotoxicity, and induction of apoptosis. RTX has been used in numerous clinical studies, including two RCTs for the treatment of both aggressive treatment-resistant and naïve ILD-SSc patients [127]. A few studies showed that RTX could increase FVC and DLCO in patients with SSc-ILD [128,129]. Bearing this in mind, rituximab has emerged as a potential first line treatment for patients with ILD-SSC [125].
- **Tocilizumab (TCZ):** TCZ is an anti-interleukin 6 (IL6) receptor monoclonal antibody. TCZ inhibition of the IL-6 receptor decreasing myofibroblast activation and reduced M2 macrophage polarization, resulting in a portion of the demonstrated antifibrotic effects of TCZ in the setting of ILD-SSc [130]. In 2018, Denton CP et al. demonstrated that regarding the exact mechanism, the effect of IL-6Rα blockade on biological pathways dominated by TGFβ-related biology is striking and highlights the interaction between IL-6 and TGFβ. Hence, IL-6 and TGFβ may form a self-sustaining loop leading to fibrosis that could be interrupted by blockade of either or both cytokines [131]. These results have also positioned tocilizumab as a first-line treatment [126].
- **Nintedanib:** Nintedanib is a tyrosine kinase inhibitor which has been shown to have antifibrotic and antiinflammatory effects in preclinical models of systemic sclerosis and ILD. In 2019 Distler, O. published the SENSCIS trial showing that the annual rate of decline of FVC in ILD-SSc patients was lower with nintedanib than with placebo. This paved the way for the use of antifibrotic drugs that were initially approved for idiopathic pulmonary fibrosis to be used in patients with ILD-SSc [132]. Currently guidelines recommend nintedanib as add on therapy to immunosuppressive treatment, but it may be reasonable to use it as a first line agent in patients with a predominantly fibrotic pattern and quiescent extra pulmonary disease, or patients who get recurrent infections on immunosuppressive therapy [126].
- **Nerandomilast:** Nerandomilast is a preferential phosphodiesterase 4B (PDE4B) inhibitor currently undergoing phase III clinical trials for idiopathic pulmonary fibrosis and progressive fibrosing interstitial lung diseases [133]. Preclinical studies have demonstrated that nerandomilast exhibits anti-inflammatory and anti-fibrotic properties in both in vitro and in vivo models of pulmonary fibrosis [134]. Compared to the pan-PDE4 inhibitor roflumilast, nerandomilast is approximately 10 times more preferential for PDE4B than for PDE4D. Inhibition of PDE4 leads to increased intracellular cAMP levels, which can impact inflammatory and immune responses, but selecting the PDE4B isoform significantly reduces side effects [135]. Nerandomilast also significantly reduced the infiltration of CD3+ T lymphocytes and F4/80+ macrophages in fibrotic areas of the lung tissue in the bleomycin-induced ILD-SSC model mice. Furthermore, nerandomilast can greatly suppress the gene expression of inflammatory cytokines including TNF-α, IL-6, IL-13, TGF-β1, IL-1β, IL-17A, and IL-13. These findings suggest that nerandomilast can modulate macrophages and T lymphocytes and suppress the gene expression of inflammatory cytokines [133]. Further studies are needed to verify the efficacy of nintedanib in patients with ILD-SSC, but it is currently considered one of the most promising therapeutic options for this disease.
- **Janus Kinase (JAK) inhibitors:** Baricitinib is a JAK1/2 inhibitor that was initially approved for the treatment of adults with moderate-to-severe rheumatoid arthritis (RA) [136]. The study by Wang et al. experimentally investigated how the JAK2 pathway inhibitor baricitinib can influence pulmonary fibrosis associated with systemic sclerosis by interacting with TGF-β1 signaling. The results of the experiment show that baricitinib reduces fibrosis in the lungs and on the skin by lowering the levels of proinflammatory factors and the expression of key components of the TGF-β1 pathway, particularly TβRI/II receptors, in animal models and in cultured human pulmonary fibroblasts. The research demonstrates significant crosstalk between the JAK2 and TGF-β1 pathways. By blocking JAK2, baricitinib interrupts the pro-fibrotic signal activated by TGF-β1. This leads to a decrease in fibroblast activation and therefore in the progression of pulmonary fibrosis in systemic sclerosis. This positions baricitinib as a potential antifibrotic therapy for systemic sclerosis-associated interstitial lung disease (SSc-ILD), acting on central molecular mechanisms in the disease [137].
- **CAR-T19 therapy:** T-cell (CAR-T19) therapy has emerged as a potential game-changer for autoimmune diseases, including SSc. CAR-T19 therapy selectively targets and depletes CD19+B cells, offering a more profound and potentially long-lasting immunomodulatory effect compared with conventional B-cell depletion therapies like RTX [138]. This approach may be particularly valuable for patients with aggressive disease phenotypes, where early intervention could prevent irreversible vasculafibrosis and organ dysfunction. Clinical improvements were accompanied by a shift to a naive B-cell phenotype, which was accompanied by reduction in autoantibody concentrations. These results support the concept that restoration of immune homoeostasis might affect the disease course in systemic sclerosis [139,140]. Although these results are promising, further studies are needed to confirm the long-term effectiveness and safety of the treatment.
- **Bispecific antibodies (bsAbs):** Preclinical models have explored bsAbs designed to engage T cells for selective depletion of autoreactive B cells (eg, CD3/CD19 or CD3/CD20 constructs), which may offer deeper and more durable immune modulation than conventional B-cell depletion alone [141]. Additionally, bispecific constructs targeting fibroblast activation protein (FAP) on pathogenic myofibroblasts have shown potential to directly reduce tissue fibrosis. While clinical application in SSc is still at an early stage, bsAbs represent an exciting frontier for future trials [140].

Finally, as Antoniou et al. suggested, patients with ILD should be closely monitored. They should be assessed holistically, and therapeutic strategies should be tailored to each individual case [142].

## 7. Conclusions

Interstitial lung disease associated with systemic sclerosis (ILD-SSc) is one of the most severe and life-threatening manifestations of this autoimmune disorder. As it has been explained, its pathophysiology results from a complex interplay between genetic predisposition, environmental factors, immune dysregulation, and the persistent activation of profibrotic signaling pathways. Central to this process is transforming growth factor beta (TGF-β), a key regulator of fibroblast activation, lung tissue remodeling, and fibrosis perpetuation.

From a medical standpoint, the detection of new autoantibodies, like anti-RNPC3, is becoming more and more important for improving the precision of diagnoses and improving the classification of outcomes in systemic sclerosis. In parallel, the integration of radiologic and histopathologic characterization with the study of molecular and epigenetic biomarkers has enhanced risk assessment and facilitated the development of more personalized therapeutic approaches.

Despite these advances, significant knowledge gaps persist. The initial molecular triggers linking immune activation to fibroblast dysfunction and chronic tissue injury are not fully understood. Further research is needed to clarify the mechanisms underlying macrophage polarization, cytokine signaling (including IL-6, IL-13, and TGF-β pathways), and endothelial-to-mesenchymal transition. Furthermore, the role of epigenetic modifications in regulating immune and fibroblast functions is a promising yet under-explored field, given their potential reversibility and significant therapeutic implications.

In summary, TGF-β remains the central orchestrator of fibrogenesis in ILD-SSc. Further studies are necessary to understand its involvement in greater depth. Novel autoantibodies, such as anti-RNPC3, enhance diagnostic and prognostic precision, and epigenetic mechanisms emerge as pivotal, potentially reversible modulators of disease progression. A more profound and interconnected comprehension of these elements is indispensable to refine preliminary diagnostic strategies, optimize prognostic modeling, and formulate targeted therapies with the potential to modify the natural progression of this devastating disease.

## Figures and Tables

**Table 1:** Summary of the most common autoantibodies in SSc (created by the authors, based on data from references [4,58,59,60,61,62,63,64,65]).

Antibody	Frequency	Clinical	References
Anticentromere (ACA)	20–30%	lcSSc, esophageal disease, PAH, digital ischemia (‘protection’ against pulmonary fibrosis)	[4,58]
Anti-topoisomerase I (Anti-Scl-70)	15–20% (40% dcSSc, 15% lcSSc)	dcSSc, ILD. Worse prognosis	[4,59]
Anti-PM-Scl	3%	Mutually exclusive with ACA. 50% of patients with polymyositis/SSc overlap	[4,61]
Anti-Th/To	2–5%	lcSSc, small joints, ILD, PAH	[4,62]
Anti-U3-RNP (fibrillarin)	4%	Myositis, PAH, renal disease. Mutually exclusive with ACA and Anti-Scl-70	[4,63]
Anti-U1-RNP	8%	lcSSc, SLE, overlap with MCTD. Poor prognosis	[4,64]
Anti-RNPC3	4.3%	lcSSc, ILD, gastrointestinal involvement, cancer. Poor prognosis	[65]

Abbreviations: SSc: Systemic sclerosis; lcSSc: Limited cutaneous systemic sclerosis; dcSSc: Diffuse cutaneous systemic sclerosis; PAH: Pulmonary arterial hypertension; ILD: Interstitial lung disease; SLE: Systemic lupus erythematosus; MCTD: Mixed connective tissue disease.

## Data Availability

No new data were created or analyzed in this study. Data sharing is not applicable to this article.
